# Microstructure in patients with visual snow syndrome: an ultra-high field morphological and quantitative MRI study

**DOI:** 10.1093/braincomms/fcac164

**Published:** 2022-06-23

**Authors:** Myrte Strik, Meaghan Clough, Emma J Solly, Rebecca Glarin, Owen B White, Scott C Kolbe, Joanne Fielding

**Affiliations:** Melbourne Brain Centre Imaging Unit, Department of Radiology, Melbourne Medical School, University of Melbourne, Melbourne, VIC 3010, Australia; Department of Neuroscience, Central Clinical School, Monash University, Melbourne, VIC 3004, Australia; Department of Neuroscience, Central Clinical School, Monash University, Melbourne, VIC 3004, Australia; Melbourne Brain Centre Imaging Unit, Department of Radiology, Melbourne Medical School, University of Melbourne, Melbourne, VIC 3010, Australia; Department of Neuroscience, Central Clinical School, Monash University, Melbourne, VIC 3004, Australia; Department of Neuroscience, Central Clinical School, Monash University, Melbourne, VIC 3004, Australia; Department of Neuroscience, Central Clinical School, Monash University, Melbourne, VIC 3004, Australia

**Keywords:** visual snow syndrome, morphometry, quantitative MRI, T1 relaxation, 7T MRI

## Abstract

Visual snow syndrome is a neurological condition characterized by continuous visual disturbance and a range of non-visual symptoms, including tinnitus and migraine. Little is known about the pathological mechanisms underlying visual snow syndrome. Here, we assessed brain morphometry and microstructure in visual snow syndrome patients using high-resolution structural and quantitative MRI. Forty visual snow syndrome patients (22 with migraine) and 43 controls underwent 7-Tesla MRI (MP2RAGE, 0.75 mm isotropic resolution). Volumetric and quantitative T1 values were extracted for white and grey matter regions and compared between groups. Where regions were significantly different between groups (false discovery rate corrected for multiple comparisons), *post hoc* comparisons were examined between patients with and without migraine. For visual snow syndrome patients, significant MRI variables were correlated with clinical severity (number of visual symptoms, perceived visual snow intensity, disruptiveness, fatigue and quality of life) and psychiatric symptoms prevalent in visual snow syndrome (depression, anxiety and depersonalization). Finally, cortical regions and individual thalamic nuclei were studied. Compared with controls, visual snow syndrome patients demonstrated a trend towards larger brain and white matter volumes and significantly lower T1 values for the entire cortex (*P* < 0.001), thalamus (*P* = 0.001) and pallidum (*P* = 0.001). For the patient group, thalamic T1 correlated with number of visual symptoms (*P* = 0.019, *r* = 0.390) and perceived disruptiveness of visual snow (*P* = 0.010, *r* = 0.424). These correlations did not survive multiple comparison corrections. As for specificity in visual snow syndrome group, T1 changes were most evident in caudal regions (occipital cortices) followed by parietal, temporal and prefrontal cortices. T1 values differed between groups for most individual thalamic nuclei. No differences were revealed between patients with and without migraine. In visual snow syndrome patients, we observed no changes in morphometry, instead widespread changes in grey matter microstructure, which followed a caudal-rostral pattern and affected the occipital cortices most profoundly. Migraine did not appear to independently affect these changes. Lower T1 values may potentially result from higher neurite density, myelination or increased iron levels in the visual snow syndrome brain. Further investigation of these changes may enhance our understanding of the pathogenesis of visual snow syndrome, ultimately leading to new treatment strategies.

## Teaser

Strik *et al*. reveal no evidence of gross brain morphometry changes in people with visual snow syndrome, but report widespread changes in grey matter microstructure, which followed a caudal-rostral pattern and affected the occipital cortices most profoundly. None of these changes were directly associated with the co-occurrence of migraine.

## Graphical Abstract

**Graphical abstract:**
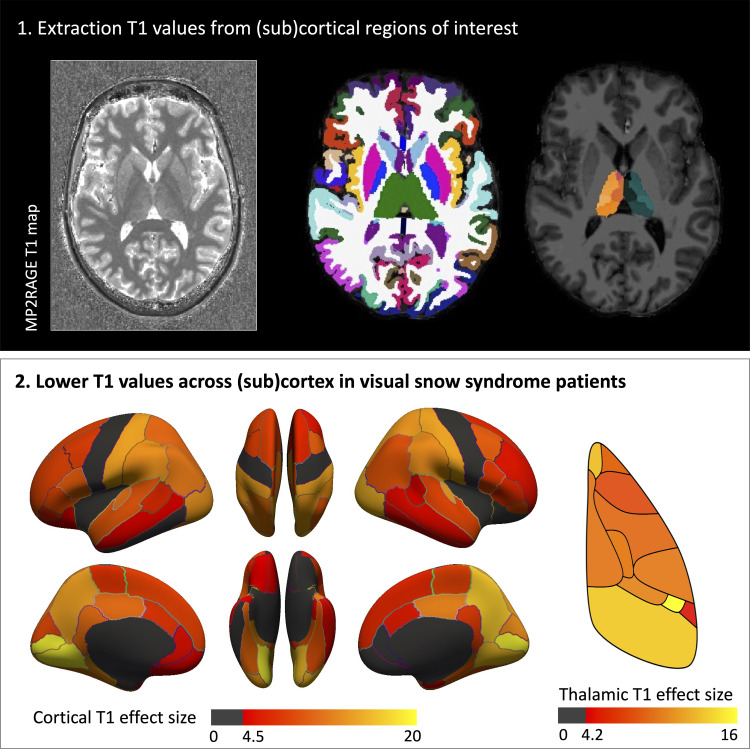

See Kondziella (https://doi.org/10.1093/braincomms/fcac178) for a scientific commentary on this article.

## Introduction

Visual snow syndrome is a neurological disorder primarily characterized by visual snow, a continuous and dynamic visual disturbance across the entire visual field. A diagnosis of visual snow syndrome requires that visual snow is accompanied by at least two other visual symptoms including after images (palinopsia), light sensitivity (photophobia), night blindness (nyctalopia) and enhanced entoptic phenomena.^1^ Patients often experience a range of non-visual sensory symptoms such as tinnitus, depersonalization and, most frequently, migraine. In addition, patients report high levels of anxiety and depression.^1,2^ Visual snow syndrome onset is commonly in young adulthood^3^ with symptoms often severely affecting daily activities^4^ and independence.^5^ Visual snow syndrome is not uncommon, with an estimated prevalence of 2% in the United Kingdom,^6^ but its underlying pathophysiology is unknown and no effective treatment options are currently available.^7^

Although there have been relatively few neuroimaging studies conducted in patients with visual snow syndrome, altered metabolism has been revealed using fluorodeoxyglucose-PET,^8,9^ and altered brain activation using visual snow-like stimuli.^8,10^ Anatomically, grey matter (GM) volume changes have been found in relatively small areas involved in vision^8,11^ as well as non-typical vision regions such as the cerebellum^11^ and limbic, temporal and parietal regions,^8^ suggesting whole brain involvement, potentially explaining the wide range of symptoms reported by patients. However, the direction of volumetric change is equivocal, with both larger^8,11^ and smaller^8^ volumes reported. Whether brain microstructure is altered in visual snow syndrome, is unknown.

We aimed to assess alterations in brain morphology and microstructure in patients with visual snow syndrome using high resolution structural imaging acquired using ultra-high field MRI [7 Tesla (7T)]. We included patients with and without migraine to ascertain the potential impact of migraine on any changes revealed. In addition to white matter (WM) and GM volumetric analyses we assessed tissue microstructure using quantitative T1 mapping. This has not been assessed previously in visual snow syndrome. Finally, we examined whether changes in volumetrics and or T1 relaxation times were related to clinical features of visual snow syndrome and severity of psychiatric symptoms. Given the wide variety of symptoms, and changes previously revealed in visual snow syndrome, we hypothesized that these patients would exhibit volumetric changes, and potentially differences in brain microstructure.

## Methods

### Participants

Forty people with visual snow syndrome (21 females; age = 33.2 ± 10.1 years) were recruited (online, television and radio), and were examined by a neurologist who confirmed their diagnosis using diagnostic criteria for snow syndrome as specified by the International Classification of Headache Disorders (Table 1). To study the effects of migraine, similar numbers of patients without a history of migraine (*n* = 17; 7 females; age = 31.6 ± 9.7 years) and with a history of migraine (*n* = 22; 14 females; age = 34.7 ± 10.5 years) were included. Patients who experienced a migraine at the time of assessment, or 3 days prior to or after testing were excluded. Additional exclusion criteria were use of medications with known effects on cognitive functioning and/or visual processing, a neurological disorder other than visual snow syndrome, and any afferent visual processing abnormalities. The latter were assessed via a full ophthalmic examination including tests of colour vision, visual acuity, retinal structure and retinal function.

**Table 1:** International Classification of Headache Disorders-3 criteria for the diagnosis of VSS

A. Dynamic, continuous, tiny dots across the entire visual field, persisting for >3 months
B. Additional visual symptoms of at least two of the following four types:
1. Palinopsia
2. Enhanced entoptic phenomena
3. Photophobia
4. Impaired night vision (nyctalopia)
C. Symptoms are not consistent with typical migraine visual aura
D. Symptoms are not better accounted for by another disorder

Forty healthy controls (25 females; age = 29.2 ± 7.6 years) were recruited through word-of-mouth advertising. No controls reported diagnosis of any neuro-ophthalmological or neurological conditions.

### Standard protocol approvals, registrations and patient consents

This study was conducted in accordance with the Declaration of Helsinki. Ethics approval was granted by Monash University Human Research Ethics Committee. All participants provided voluntary, written informed consent prior to participation.

### Clinical assessments

For visual snow syndrome patients, visual symptoms (after images, nyctalopia, photophobia, floaters, blue field entopic phenomenon, self-light of the eye and halos) and other sensory symptoms (tinnitus, paraesthesia, tremors and dizziness) were recorded. Patients also self-rated their visual snow intensity (low to extreme, 1–6), disruptiveness (not at all to severely, 1–7), and impact on quality of life (not at all to severely, 1–7). As an exploratory analysis psychiatric symptoms were studied. Self-rating scale questionnaires including the Depression Anxiety Stress Scale, Fatigue Severity Scale and Cambridge Depersonalization Scale were sent after the session and four patients did not respond.

### Imaging acquisition

All participants were scanned using a whole-body Siemens MAGNETOM 7T MRI system (Siemens Healthcare, Erlangen, Germany) with a combined single-channel transmit and 32-channel receive head coil (Nova Medical, Wilmington, MA, USA). High-resolution anatomical imaging was acquired using a three-dimensional T_1_-weighted sequence (MP2RAGE) with the following imaging parameters: repetition time = 5000 ms, echo time = 3.06 ms, inversion time = 700/2700 ms, flip angle = 4°/5°, 224 slices, acceleration factor = 4, phase encoding direction = anterior-to-posterior, voxel size = 0.75 mm isotropic, image matrix = 330 × 330, imaging plane = sagittal. From the acquired MP2RAGE, a denoised uniform T1 weighted image and T1 map were derived for volumetric and T1 measurements respectively.

### Anatomical MRI processing

**Figure 1:**
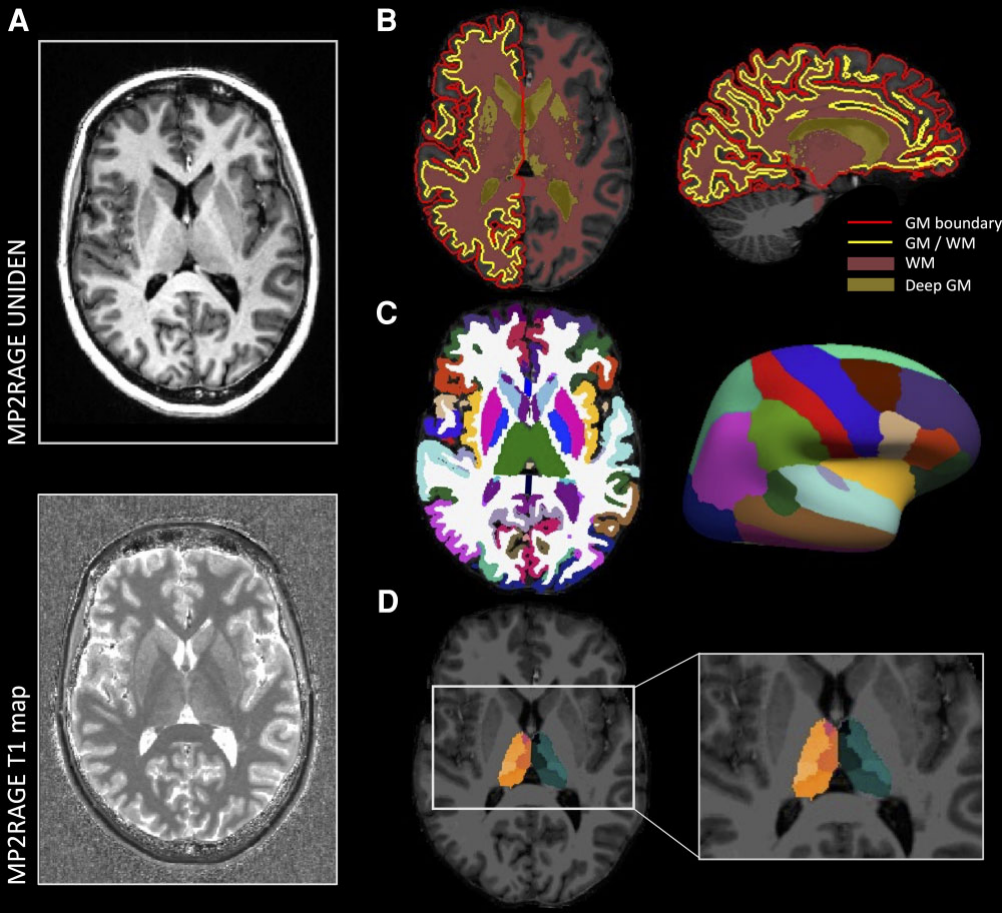
**Anatomical processing pipeline.** (**A**) From the MP2RAGE image, a uniform denoised image (UNIDEN) and T1 map were acquired. (**B**) The UNIDEN image was used for segmentation of GM, deep GM, WM as well as parcellations of (**C**) cortical and subcortical regions and (**D**) thalamic nuclei. From these (sub)cortical parcellations, volumetrics were calculated and T1 values were extracted.

The uniform denoised MP2RAGE images were used for cortical and subcortical segmentation analyses using FreeSurfer (version 6.0-patch, https://surfer.nmr.mgh.harvard.edu/) (Fig. 1). Brain segmentations were visually inspected and were manually edited, if needed, including removal of brain masks and or priming of WM. Regions of interest (ROI) included WM, cortical GM, thalamus, putamen, pallidum, caudate nucleus and cortical surface parcellations (68 regions). Thalamic nuclei were segmented using FreeSurfer (version 7.0) (Fig. 1).

For each participant and each ROI, volumes were derived from the FreeSurfer statistical output (asag.stats or lh/rh.aparc.stats) and T1 values were extracted (Fig. 1) using a customized MATLAB R2019b (Mathworks, Natick, MA, USA) script. To prevent possible introduction of noise caused by inaccuracy in segmentations with cerebrospinal fluid near the surface or GM and WM boundaries, T1 values were extracted from the midpoint between the GM/WM and pial surfaces. To determine whether this measure was representative we also sampled from 11 equally distant points between each GM/WM and matching pial surface vertex and T1 values were extracted and averaged across each ROI using a customized MATLAB R2019b script (Mathworks, Natick, MA, USA).

Due to B0 and B1 inhomogeneities, the cerebellum could not be reliably segmented and was therefore excluded from analyses. Similarly, T1 values could not be reliably extracted for paratenial, paracentral, ventromedial thalamic nuclei and were therefore excluded from both volumetric and quantitative analyses.

### Statistical analyses

Demographic and clinical variables were compared between groups (controls, visual snow syndrome patients) using independent samples *t*-tests, or Mann–Whitney U-tests for non-normally distributed data and χ^2^ test for nominal variables using SPSS Statistics (version 27, IBM, USA).

Brain volumetrics and quantitative T1 measures of the WM, cortical and deep GM (pallidum, putamen, caudate nucleus and thalamus) structures were compared between groups (controls, visual snow syndrome patients) using general linear models (dependent variables MRI metrics, fixed factor groups). Intracranial volume was included as a covariate in volumetric analyses. Significant MRI measures (false discovery rate corrected for multiple comparisons) were further compared between visual snow syndrome patients with and without migraine and correlated with clinical variables. Linear regressions were performed with significant measures as dependent variables and age or disease duration as an independent variable to examine potential age and disease duration effects. Saved residuals were used in correlation analyses with clinical and psychiatric variables using Pearson correlations for normally distributed data and Spearman for non-normally distributed data.

To assess specificity of these results, cortical regions, cortical layers and individual thalamic nuclei were studied separately and compared between groups (controls, visual snow syndrome patients), with significant variables further compared between visual snow syndrome patients with and without migraine.

For all analyses, multiple comparisons corrections were performed using a false discovery rate with a corrected false-positive probability of <0.05.^12^

### Data availability

Data in this article can be made available by the corresponding author on reasonable request.

## Results

### Demographics and clinical variables

Demographics, clinical self-ratings and questionnaire scores are summarized in Table 2. Visual snow syndrome patients did not differ in age or sex from controls. Compared with patients without migraine, patients with migraine reported lower visual snow intensity (*P* = 0.020, *p_FDR_* > 0.05), but this did not survive false discovery rate correction.

**Table 2:** Demographics and clinical characteristics

	Controls	VSS		VSS without migraine	VSS with migraine	
**Demographics**			*P*-value			*P*-value
Participant, *n*	43	40		17	22	
Sex, F/M	25/18	21/19	0.117	7/10	14/8	0.322
Age, years	29.24 (7.60)	33.18 (10.08)	0.662	31.55 (9.69)	34.69 (10.54)	0.206
**Visual snow syndrome**						
Family history VSS, y/n		1/35		0/15	1/20	
Family history migraine, y/n		20/16		5/10	15/6	
Lifelong VSS, y/n		15/21		6/9	9/12	
Disease duration, years		20.10 (12.65)		18.59 (12.25)	20.96 (13.33)	0.573
Visual symptoms^a^ (of 8)		5.33 (1–8)		5.13 (1–8)	5.48 (3–8)	0.858
Sensory symptoms^a^ (of 4)		1.36 (0–4)		1.53 (0–3)	1.24 (0–4)	0.343
**VSS self-ratings**						
Intensity^a^ (1–6)		4.03 (1–6)		4.53 (2–6)	3.67 (1–6)	0.020
Disruptiveness^a^ (1–7)		3.44 (1–7)		4.00 (1–7)	3.05 (1–5)	0.154
Impact quality of life^a^ (1–7)		3.58 (1–7)		3.67 (1–7)	3.52 (1–7)	0.909
**DASS**						
Anxiety		7.89 (7.59)		8.67 (8.49)	7.33 (7.04)	0.498
Depression		5.36 (5.30)		4.87 (5.68)	5.71 (5.13)	0.710
Stress		9.86 (7.56)		9.4 (6.91)	10.19 (8.15)	0.859
Overall DASS		23.11 (17.56)		22.93 (17.88)	23.24 (17.76)	0.710
**CSD depersonalization**		46.68 (37.90)		40.3 (39.58)	50.22 (37.63)	0.472
**FSS fatigue**		36.86 (13.08)		35.87 (14.14)	37.57 (12.58)	0.596

CSD = Cambridge Depersonalization Scale; DASS = Depression Anxiety Stress Scale; F = Female; FSS = Fatigue Severity Scale; M = Male; n = no; VSS = visual snow syndrome; y = yes.

Values are mean scores and standard deviations unless indicated otherwise.

^a^ Mean and range.

### WM and GM regions

Volumetric and quantitative T1 values for WM, cortical and deep GM for each group are summarized in Table 3.

**Table 3:** Brain volumetrics and quantitative T1 values

	Controls (*n* = 43)	VSS (*n* = 40)		VSS without migraines (*n* = 17)	VSS with migraines (*n* = 22)	
Volumetrics (mm^3^)			*P*-values			*P*-values
Brain^a^	1197.88 (77.41)	1233.76 (98.27)	**0**.**013**	1249.12 (101.65)	1220.36 (98.13)	0.129
Cortical GM^a^	474.67 (40.83)	478.30 (46.17)	0.563	485.92 (43.37)	471.22 (48.92)	0.429
WM^a^	318.6 (34.96)	337.59 (53.11)	**0**.**021**	342.02 (56.86)	334.822(52.33)	0.064
Thalamus^a^	13.66 (1.08)	13.76 (1.44)	0.615	13.90 (1.16)	13.61 (1.65)	0.280
Pallidum	3.68 (0.4)	3.8 (0.4)	0.056	3.9 (0.4)	3.8 (0.4)	0.358
Putamen	9.70 (0.9)	9.7 (1.1)	0.954	9.7 (0.8)	9.6 (1.3)	0.978
Caudate nucleus	7.29 (0.8)	7.3 (1.0)	0.755	7.5 (0.9)	7.2 (1.0)	0.558
**T1 values (ms)**						
Cortical GM	1885.26 (40.43)	1852.88 (37.18)	**<0**.001*	1856.71 (39.44)	1848.74 (36.33)	0.517
WM	1354.24 (89.11)	1317.34 (76.18)	**0**.**047**	1308.92 (65.75)	1320.69 (84.77)	0.639
Thalamus	1428.84 (51.55)	1393.66 (34.73)	**0**.001*	1397.62 (37.88)	1389.48 (32.984)	0.478
Pallidum	1201.70 (36.03)	1175.26 (31.67)	**0**.001*	1183.99 (37.36)	1168.15 (26.10)	0.127
Putamen	1501.07 (39.33)	1480.71 (48.89)	**0**.**039**	1490.39 (55.88)	1471.62 (42.88)	0.242
Caudate nucleus	1711.52 (92.44)	1675.83 (80.70)	0.065	1669.71 (58.45)	1677.62 (96.22)	0.767

All values represent mean scores and standard deviations unless denoted otherwise. *P*-values are marked in bold.

^a^ Mean and standard deviations are noted in 10^3^.

* *P*-values that survived false discovery rate multiple comparison correction.

**Figure 2:**
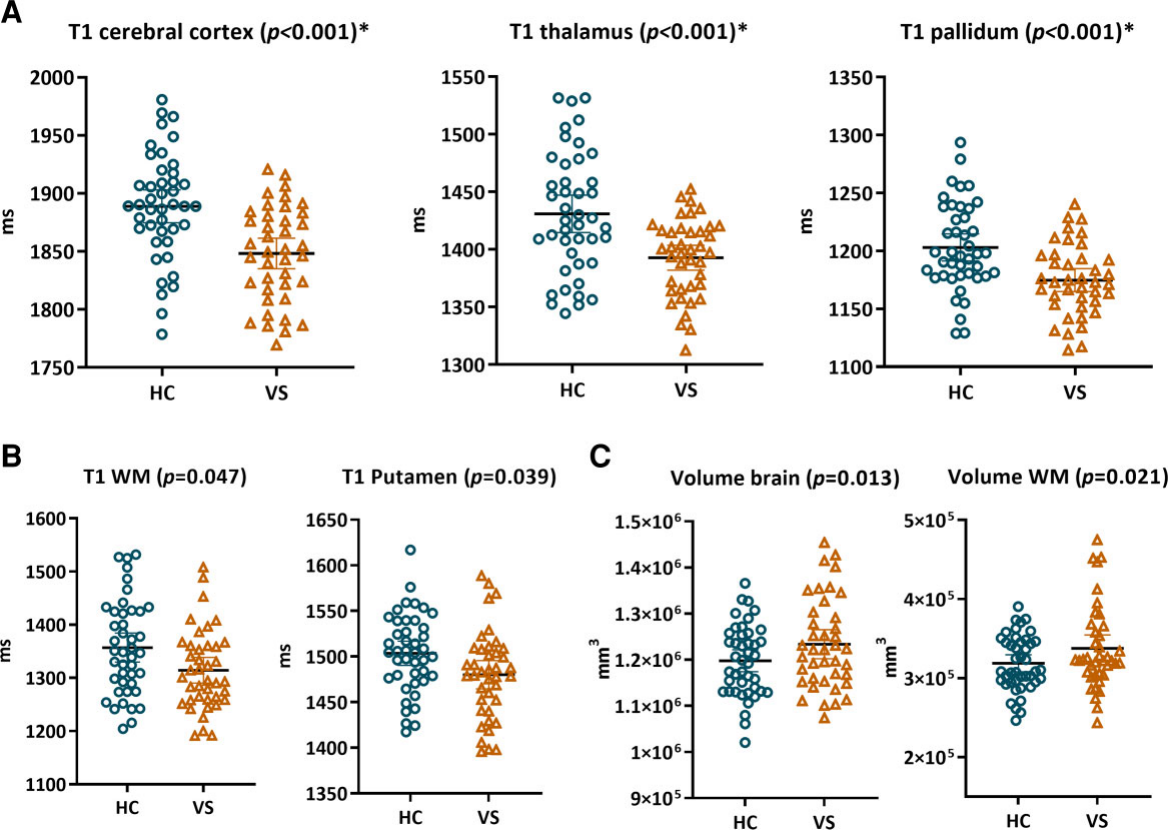
**Brain volumetrics and quantitative T1 value group differences.** Brain volumetrics and quantitative T1 measures were compared between groups (controls and visual snow syndrome patients) using general linear models (dependent variables MRI metrics, fixed factor groups). **(A**) Visual snow syndrome patients demonstrated significantly lower T1 values for the entire cerebral cortex, thalamus and pallidum (*p*_FDR_ < 0.05), compared with controls. (**B**) Shorter WM and putamen T1 values and (**C**) larger brain and WM volumes were found, but these comparisons did not survive multiple comparison corrections.

As for the group differences, brain volumetrics revealed a trend towards larger brain (*P* = 0.013) and WM volumes (*P* = 0.021) for visual snow syndrome patients, but this did not survive multiple comparison correction (Fig. 2). Quantitative T1 values were significantly lower for entire cortical GM (*P* < 0.001), thalamus (*P* = 0.001), pallidum (*P* = 0.001), putamen (*P* = 0.039, *p_FDR_* > 0.05) and WM (*P* = 0.047, *p_FDR_* > 0.05) for patients relative to controls (Fig. 2).

These significant variables were further compared between visual snow syndrome patients with and without migraine and correlated with clinical variables. No differences were observed in T1 values between patients with and without migraine. In the visual snow syndrome group, thalamic T1 correlated with the number of visual symptoms (*r* = 0.390, *P* = 0.019, *p*_FDR_ > 0.05) and patient subjective ratings of visual snow disruptiveness (*r* = 0.424, *P* = 0.010, *p*_FDR_ > 0.05), but these did not survive multiple comparison correction. After correcting for disease duration instead of age, similar correlations were found as well as a correlation between pallidum T1 values and the number visual symptoms (*r* = 0.343, *P* = 0.040, *p*_FDR_ > 0.05).

### Cortical and thalamic specificity

No significant differences in volumetrics were observed between visual snow syndrome patients and controls. Compared to controls, visual snow syndrome patients’ volumetric data revealed only trends towards a larger left insula (*P* = 0.008), middle temporal cortex (*P* = 0.040) and superior frontal region (*P* = 0.040), a smaller left pars opercularis (*P* = 0.001) and fusiform (*P* = 0.007) volume and larger thalamic nuclei, including LD (*P* = 0.003) and LP (*P* = 0.012). None survived false discovery rate correction.

**Figure 3:**
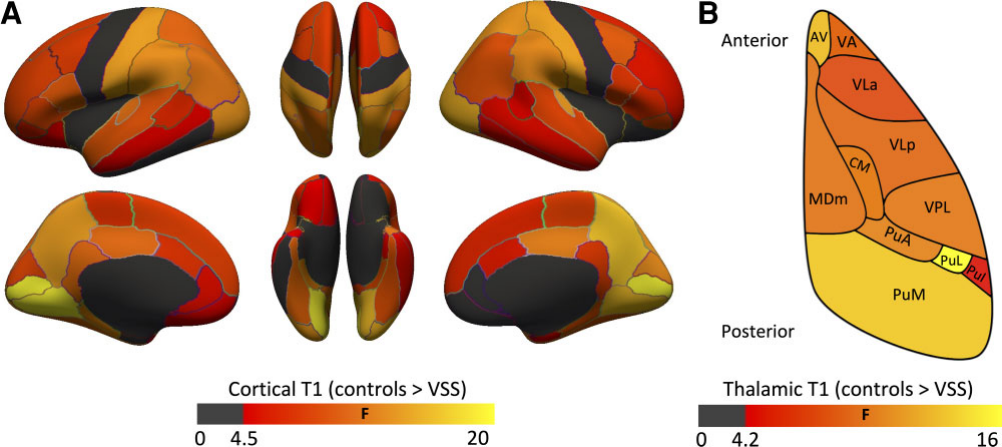
(**A**) Compared to controls, in VSS patients a notable caudal to rostral gradient was observed in the strength of effect with occipital regions that showed the greatest difference, followed by parietal, temporal and prefrontal cortices. No differences were observed in inferior temporal regions, frontal pole, orbitofrontal cortex, insula and precentral gyrus. (**B**) Although less striking compared to cortex, the thalamus also displayed a caudal [posterior (P)) to rostral (anterior (A)] effect size gradient with strongest effect in lateral pulvinar (PuL). AV = anteroventral; CM = centromedian; MDm = mediodorsal medial magnocellular; PuA = pulvinar anterior; PuI = pulvinar inferior; PuM = pulvinar medial; VA = ventral anterior; VLa = ventral lateral anterior; VLp = ventral lateral posterior; VPL = ventral posterolateral nucleus.

Lower T1 values were revealed for visual snow syndrome patients for most cortical regions (*p*_FDR_ < 0.05) (Fig. 3, Supplementary Tables 1 and 2) relative to controls. Effect sizes display a caudal-rostral pattern, with caudal areas more strongly affected. No between-group differences were revealed in the inferior temporal cortex, frontal pole, orbitofrontal cortex, insula and precentral gyrus. Lower T1 values were observed for most thalamic nuclei (*p*_FDR_ < 0.05) (Fig. 3, Supplementary Tables 3 and 4). Of these significant variables, none significantly differed between visual snow syndrome patients with and without migraine (Supplementary Tables 1–4). T1 differed in most cortical layers (Supplementary Fig. 1).

## Discussion

This study investigated brain morphometry and microstructure in patients with visual snow syndrome using ultra-high field 7 T MRI. We found widespread alterations to GM microstructure in the absence of gross differences in morphometry in visual snow syndrome patients relative to controls. No independent effect of migraine was observed. Changes in microstructure appear to be relatively non-specific, but a rostro-caudal pattern was observed with change most evident in caudal regions including occipital cortices.

### Widespread changes in GM microstructure

In patients with visual snow syndrome, widespread changes were revealed in the microstructural composition of GM, evident as a reduction in T1 relaxation times relative to controls. T1 relaxation time is associated with recovery of longitudinal magnetization after a radiofrequency pulse, and has been proposed to relate to various molecular and microstructural features of brain tissue including water content in intra/extracellular and vascular subspaces, the abundance of macromolecules in neurites and myelin, and the presence of iron.^13^ Quantitative T1 mapping has been used as a proxy for microstructural changes in brain diseases such as multiple sclerosis,^14–16^ where increased T1 has been reported in the context of glial and neuronal damage^14,15^ and in cortical GM.^16^ Here, we observed shorter GM T1 relaxation times in visual snow syndrome patients, which could therefore result from higher neurite density and/or myelination, reduced water content or increased iron levels. We observed a trend towards lower T1 values in the WM in visual snow syndrome patients, which is consistent with a recent diffusion-weighted imaging study that reported higher fractional anisotropy in the WM of visual and non-visual regions in patients, interpreted as evidence of higher excitability.^17^

While no causal claims can be made, both increased myelination and neurite density could be associated with hyperexcitability, a commonly proposed hypothesis in the visual snow syndrome literature that suggests widespread dysfunction of higher-order cortical processes.^4,18,19^ Another hypothesis suggests that visual snow syndrome could be related to disruption within or between the thalamus, an important sensory relay centre, and the cortex: thalamocortical dysrhythmia.^20^ This theory has been proposed to explain the connectivity and electrophysiological changes found in visual snow syndrome patients.^21^ Given the rich interconnectedness and widespread connectivity within the thalamic nuclei, we investigated these nuclei separately and found that the microstructural composition of the entire thalamus is affected in visual snow syndrome. To explore the hypotheses of cortical hyperexcitability and thalamocortical dysrhythmia more fully, future studies might combine structural and functional imaging. Particularly investigating the connectivity and network topology might lead to more insight into the functional mechanisms underlying visual snow syndrome.

Regions of altered T1 in visual snow syndrome patients included most cortical regions and thalamic nuclei, with the abnormality relatively consistent across cortical layers. However, there was an observable pattern in the degree of change, with a notable caudal-rostral pattern. The greatest differences were observed in occipital regions (particularly pericalcarine cortex which includes the primary visual cortices), followed by parietal cortex, with significant but less evident changes in the temporal and prefrontal cortices. Interestingly, we observed no difference in either the left or right precentral gyrus (primary motor cortex) despite significant differences in the strongly connected postcentral gyrus (primary somatosensory cortex). The lack of primary motor cortex differences is in line with the wide variety of visual and secondary sensory symptoms and lack of motor disabilities experienced by people with visual snow syndrome.^2^

Despite the caudal-rostral pattern, microstructural changes appear to be a relatively non-specific disease feature. This non-specificity is consistent with the wide variety of symptoms observed in visual snow syndrome where patients not only experience visual snow but other visual and non-visual sensory symptoms including migraine, paraesthesia and tinnitus. However, higher rather than lower T1 values correlated with a higher number of self-reported visual symptoms and perceived visual snow disruptiveness, which is somewhat counter-intuitive given our primary finding of reduced T1 in visual snow syndrome patients. One explanation might be that while the physiological aspect of the disease leads to T1 shortening in the short term, visual snow syndrome chronicity might confer deleterious effects on neurons and glia over time. Correcting for disease duration resulted in similar relations. However, whether visual snow syndrome progresses is currently unclear, with no longitudinal studies currently conducted to determine long-term sequelae. Importantly, the correlations revealed here, did not survive multiple comparisons and previous research has not found any relationship between structural and clinical measures.^11^ Together this might suggest that any relationship between brain structure and clinical visual snow syndrome characteristics is only moderate or merely a consequence of the sensitivity of rating scales, subjectiveness or statistical power.

### Brain morphometry in visual snow syndrome

In contrast to previously published findings,^8,11^ we observed a trend only, towards larger volumes of the entire brain, WM, left insula, middle temporal cortex, superior frontal region and a smaller left pars opercularis and fusiform in visual snow syndrome patients. Two previous studies have reported altered cortical volumes in visual regions such as the primary and secondary visual cortices,^11^ extrastriate visual cortex^8^ and classically non-visual regions such as cerebellum,^11^ limbic and temporal lobules.^8^ However, results from these studies are conflicting, with both increased and decreased volumes reported, and areas of change relatively small. Given the widespread and more consistent GM T1 results in this study, we argue that quantitative imaging is a more appropriate means of capturing structural changes in visual snow syndrome.

### Visual snow syndrome and migraines: overlapping pathology or distinct brain mechanisms

Migraine is a common co-morbidity in visual snow syndrome. We, therefore, studied the potential effect of migraine alone on the morphometry and microstructure of visual snow syndrome brains. In chronic migraine patients^22^ and migraineurs,^23^ previous research has found both increased^23^ and decreased^22,24^ GM volumetrics as well as altered WM integrity measured using diffusion imaging.^25^ However, we did not observe any migraine-related group differences, suggesting that microstructural changes are visual snow syndrome specific. This is in line with previous research, where statistical correction for the presence of migraine resulted in less strong but similar diffusion effects in the WM.^17^ Clinically, visual snow has been reported distinct from migraine,^1^ but migraine has been related to an increased likelihood of additional visual symptoms^9^ and a more severe presentation of the disease.^26^ In this study, patients with migraine did not have a more severe clinical phenotype, which suggests that the lack of anatomical differences between migraine and non-migraine patients are not confounded by clinical severity. In a larger group of patients including those studied in this article, migraine patients reported more visual symptoms on average.^27^

### Study limitations and future directions

Future studies employing additional quantitative imaging techniques such as myelin water or susceptibility-weighted imaging^28^ might assist in better elucidating specific features of microstructural abnormalities in visual snow syndrome. We did not analyse the cerebellum because we could not accurately segment it due to suboptimal segmentation due to B0 and B1 inhomogeneities. This study did not include a non-visual snow syndrome control group with migraine. It remains unclear whether the aetiology of migraine is consistent with that of visual snow syndrome or is associated with migraine. Future studies will be required to explore the factors involved in migraine in people with visual snow syndrome specifically.

## Conclusion

It is clear that visual snow syndrome is a disorder of the central nervous system. However, the underlying pathophysiological mechanisms remains elusive. Here, we reveal no evidence of gross morphometry changes in the visual snow syndrome brain, but widespread changes in the microstructure of the GM, the most notable of these occurring in caudal regions including the occipital cortex. None of these changes are directly associated with the co-occurrence of migraine. While we were unable to determine the specific brain tissue that underlies microstructural changes, they do focus further investigations, contributing significantly to our understanding of visual snow syndrome.

## Supplementary Material

fcac164_Supplementary_Data

## Abbreviations

GM: grey matter
ROI: region of interest
WM: white matter
