# Supplementary material for: Microstructure in patients with visual snow syndrome: an ultra-high field morphological and quantitative MRI study
Source: Brain Commun. 2022 Jun 23;4(4):fcac164. doi: 10.1093/braincomms/fcac164 (PMC9373960; doi:10.1093/braincomms/fcac164)
Supplement: fcac164_Supplementary_Data [file fcac164_Supplementary_Data.docx]

**Supplementary material**


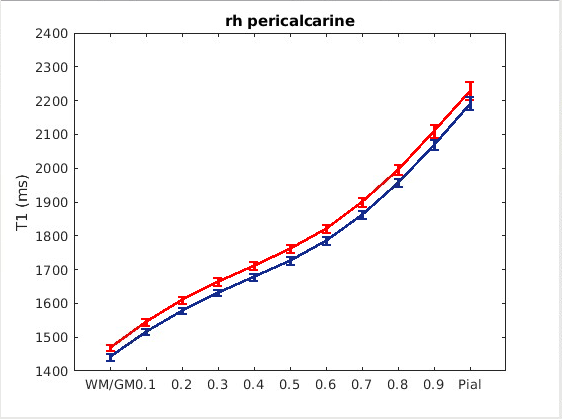

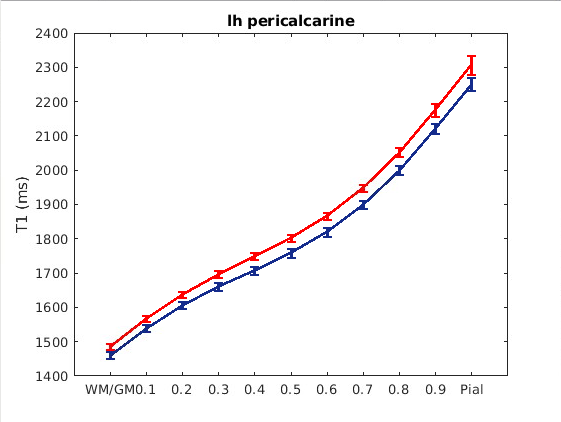


**Supplementary Figure 1. T1 values in cortical layers.** These plots visualize T1 values (mean +/- 95% confidence interval) for several layers in the pericalcarine in the left (lh) and right (rh) hemisphere for controls (in red) and visual snow syndrome patients (in blue).There where the confidence intervals do not overlap, the means are different at a significance level of p<0.05.

| **Volumes (mm^3^)** | **Controls** | **n = 43** | **VSS** | **n = 40** |  | **VSS NM** | **n = 17** | **VSS M** | **n = 22** |  |
| --- | --- | --- | --- | --- | --- | --- | --- | --- | --- | --- |
|  | Mean | SD | Mean | SD | *p*-values | Mean | SD | Mean | SD | *p*-values |
| **Left hemisphere** |  |  |  |  |  |  |  |  |  |  |
| bankssts | 2335.33 | 470.25 | 2178.60 | 485.85 | 0.133 | 2270.59 | 519.36 | 2101.73 | 468.02 | 0.531 |
| caudalanteriorcingulate | 1874.86 | 415.59 | 1835.93 | 405.09 | 0.681 | 1900.65 | 368.49 | 1755.32 | 411.73 | 0.828 |
| caudalmiddlefrontal | 6627.12 | 1220.87 | 6720.73 | 1132.36 | 0.665 | 6423.82 | 1103.97 | 6928.09 | 1151.13 | 0.021 |
| cuneus | 3552.79 | 475.50 | 3741.78 | 563.96 | 0.083 | 3741.24 | 629.99 | 3723.05 | 528.63 | 0.224 |
| entorhinal | 1476.95 | 397.67 | 1442.93 | 326.56 | 0.680 | 1406.65 | 317.66 | 1448.68 | 329.58 | 0.475 |
| fusiform | 7257.79 | 836.67 | 7471.63 | 926.14 | 0.248 | 7538.41 | 937.96 | 7379.50 | 933.96 | 0.724 |
| inferiorparietal | 13716.65 | 1800.54 | 12499.98 | 2377.67 | 0.007 | 12922.94 | 2879.89 | 12181.68 | 1981.75 | 0.878 |
| inferiortemporal | 7968.70 | 1561.23 | 7788.88 | 1307.93 | 0.580 | 7887.94 | 1189.27 | 7711.45 | 1443.99 | 0.856 |
| isthmuscingulate | 2740.74 | 507.25 | 2816.93 | 565.22 | 0.463 | 2859.94 | 543.25 | 2759.91 | 592.04 | 0.558 |
| lateraloccipital | 13223.05 | 1265.90 | 14096.95 | 1765.30 | 0.008 | 14636.76 | 1640.02 | 13705.91 | 1823.83 | 0.306 |
| lateralorbitofrontal | 7446.21 | 925.61 | 7754.73 | 976.69 | 0.077 | 7883.71 | 935.77 | 7619.18 | 1020.79 | 0.653 |
| lingual | 6805.72 | 962.14 | 7142.30 | 881.71 | 0.091 | 7139.53 | 949.69 | 7087.41 | 823.69 | 0.409 |
| medialorbitofrontal | 4606.60 | 685.73 | 4689.35 | 850.11 | 0.481 | 4774.00 | 1007.50 | 4598.91 | 734.82 | 0.206 |
| middletemporal | 10655.56 | 1595.12 | 10470.48 | 1503.67 | 0.601 | 11080.41 | 1488.55 | 9952.77 | 1370.87 | 0.083 |
| parahippocampal | 1817.23 | 240.46 | 1869.83 | 304.13 | 0.382 | 1907.06 | 337.64 | 1829.00 | 280.54 | 0.332 |
| paracentral | 3881.05 | 570.13 | 4180.90 | 887.15 | 0.040 | 4334.24 | 874.86 | 4070.09 | 919.37 | 0.937 |
| pars opercularis | 5082.53 | 902.70 | 4905.50 | 827.09 | 0.362 | 4807.82 | 818.23 | 4960.05 | 859.87 | 0.413 |
| pars orbitalis | 2613.98 | 346.69 | 2722.63 | 458.75 | 0.168 | 2812.71 | 372.88 | 2654.14 | 522.88 | 0.992 |
| pars triangularis | 4448.23 | 624.21 | 3972.28 | 651.13 | 0.001 | 3848.94 | 513.79 | 4077.00 | 748.59 | 0.218 |
| pericalcarine | 2321.63 | 495.07 | 2470.58 | 436.66 | 0.132 | 2588.24 | 487.77 | 2383.45 | 391.60 | 0.578 |
| postcentral | 10710.35 | 1429.59 | 11100.38 | 1963.00 | 0.261 | 11361.41 | 2436.44 | 10901.95 | 1592.73 | 0.999 |
| posteriorcingulate | 3406.47 | 462.13 | 3390.65 | 564.26 | 0.920 | 3535.82 | 536.74 | 3284.27 | 584.90 | 0.586 |
| precentral | 14472.44 | 1701.48 | 14660.28 | 1850.31 | 0.551 | 14832.53 | 2041.71 | 14523.00 | 1771.42 | 0.446 |
| precuneus | 10270.40 | 1236.47 | 10336.53 | 1448.87 | 0.771 | 10737.00 | 1540.83 | 9999.09 | 1350.00 | 0.624 |
| rostralanteriorcingulate | 2514.84 | 563.52 | 2596.70 | 573.74 | 0.472 | 2714.82 | 682.74 | 2488.82 | 475.76 | 0.639 |
| rostralmiddlefrontal | 16723.07 | 2650.34 | 16660.48 | 2201.10 | 0.946 | 16730.35 | 1288.04 | 16514.23 | 2740.62 | 0.322 |
| superiorfrontal | 24971.37 | 3072.07 | 25508.53 | 3013.95 | 0.307 | 25797.41 | 2599.98 | 25209.73 | 3380.38 | 0.231 |
| superiorparietal | 14415.91 | 1881.68 | 14772.95 | 2725.11 | 0.436 | 15394.59 | 2954.00 | 14292.64 | 2566.83 | 0.704 |
| superiortemporal | 11639.93 | 1453.69 | 12225.68 | 1319.94 | 0.040 | 12258.00 | 972.51 | 12178.27 | 1581.20 | 0.683 |
| supramarginal | 11847.70 | 2077.88 | 12252.40 | 2622.42 | 0.375 | 12520.12 | 3061.52 | 11927.32 | 2267.09 | 0.731 |
| frontalpole | 1148.70 | 188.84 | 1148.60 | 191.84 | 0.980 | 1158.24 | 131.71 | 1129.09 | 226.50 | 0.758 |
| temporalpole | 1534.44 | 303.98 | 1544.85 | 275.65 | 0.837 | 1520.41 | 290.59 | 1543.95 | 259.36 | 0.526 |
| transversetemporal | 1389.88 | 256.46 | 1401.50 | 250.32 | 0.806 | 1361.59 | 184.81 | 1421.59 | 293.21 | 0.114 |
| insula | 7190.26 | 803.06 | 7185.05 | 786.98 | 0.954 | 7369.41 | 801.16 | 7049.32 | 783.14 | 0.874 |
| **Right hemisphere** |  |  |  |  |  |  |  |  |  |  |
| bankssts | 2047.09 | 373.99 | 1975.93 | 474.11 | 0.456 | 1951.00 | 517.00 | 1991.91 | 461.28 | 0.404 |
| caudalanteriorcingulate | 2095.72 | 472.40 | 2271.78 | 482.05 | 0.078 | 2406.35 | 472.67 | 2140.59 | 459.99 | 0.184 |
| caudalmiddlefrontal | 6596.79 | 1296.40 | 6563.10 | 1665.25 | 0.935 | 6474.12 | 1544.59 | 6529.59 | 1756.02 | 0.704 |
| cuneus | 3927.37 | 599.08 | 4183.00 | 797.51 | 0.084 | 4216.35 | 871.81 | 4176.32 | 769.90 | 0.223 |
| entorhinal | 1727.37 | 586.05 | 1615.80 | 511.80 | 0.363 | 1642.53 | 591.56 | 1599.18 | 467.53 | 0.708 |
| fusiform | 7543.63 | 940.23 | 7672.00 | 937.04 | 0.536 | 7695.12 | 1032.59 | 7703.09 | 872.69 | 0.949 |
| inferiorparietal | 15871.56 | 2128.85 | 14986.80 | 2720.80 | 0.093 | 15037.71 | 3674.49 | 14862.27 | 1809.74 | 0.448 |
| inferiortemporal | 7298.65 | 1331.83 | 6726.78 | 1151.06 | 0.042 | 6822.88 | 1107.63 | 6672.41 | 1227.26 | 0.567 |
| isthmuscingulate | 2579.42 | 453.67 | 2627.75 | 490.64 | 0.598 | 2794.88 | 463.91 | 2488.55 | 487.69 | 0.305 |
| lateraloccipital | 13345.49 | 1701.26 | 14070.53 | 2274.71 | 0.092 | 13924.88 | 1754.61 | 14271.45 | 2650.33 | 0.241 |
| lateralorbitofrontal | 6313.40 | 946.04 | 6412.48 | 1026.95 | 0.515 | 6505.65 | 1078.62 | 6329.77 | 1027.30 | 0.231 |
| lingual | 7763.14 | 1068.32 | 8063.28 | 1151.26 | 0.200 | 7969.18 | 1304.12 | 8031.55 | 951.86 | 0.354 |
| medialorbitofrontal | 4548.53 | 760.94 | 4606.85 | 571.35 | 0.621 | 4586.06 | 569.72 | 4579.00 | 559.76 | 0.288 |
| middletemporal | 9903.37 | 1559.89 | 9368.48 | 1782.21 | 0.111 | 9457.12 | 1739.27 | 9286.50 | 1891.14 | 0.186 |
| parahippocampal | 1731.91 | 332.40 | 1774.70 | 268.20 | 0.516 | 1795.53 | 244.71 | 1754.86 | 294.75 | 0.703 |
| paracentral | 4329.05 | 674.90 | 4578.95 | 960.47 | 0.109 | 4554.00 | 1038.74 | 4577.77 | 938.61 | 0.134 |
| pars opercularis | 4323.00 | 729.15 | 4157.30 | 683.06 | 0.294 | 4105.00 | 679.25 | 4220.36 | 704.82 | 0.664 |
| pars orbitalis | 2989.05 | 491.04 | 3066.30 | 588.50 | 0.429 | 3156.71 | 515.85 | 2997.27 | 654.84 | 0.843 |
| pars triangularis | 4799.74 | 849.53 | 4750.68 | 892.79 | 0.819 | 4982.47 | 860.22 | 4593.95 | 914.18 | 0.431 |
| pericalcarine | 2718.30 | 969.22 | 2826.38 | 457.82 | 0.514 | 2953.53 | 567.41 | 2721.73 | 344.18 | 0.434 |
| postcentral | 10083.07 | 1396.56 | 10703.05 | 2350.20 | 0.111 | 11042.18 | 2568.13 | 10418.00 | 2245.53 | 0.915 |
| posteriorcingulate | 3462.35 | 433.55 | 3487.95 | 505.94 | 0.713 | 3626.12 | 509.42 | 3349.91 | 468.72 | 0.751 |
| precentral | 14129.49 | 1898.21 | 14155.85 | 1830.28 | 0.919 | 13770.06 | 1548.92 | 14424.82 | 2043.89 | 0.045 |
| precuneus | 10690.53 | 1551.19 | 10902.95 | 1566.88 | 0.441 | 11133.94 | 1321.77 | 10701.23 | 1769.22 | 0.719 |
| rostralanteriorcingulate | 1820.63 | 390.54 | 1720.50 | 446.32 | 0.274 | 1701.53 | 506.86 | 1733.64 | 416.76 | 0.179 |
| rostralmiddlefrontal | 16660.88 | 2811.71 | 16993.10 | 2410.64 | 0.451 | 17342.47 | 2407.32 | 16685.36 | 2479.09 | 0.413 |
| superiorfrontal | 23918.60 | 3143.72 | 24061.48 | 3315.79 | 0.778 | 23554.18 | 3161.37 | 24326.50 | 3485.70 | 0.040 |
| superiorparietal | 13921.28 | 2108.63 | 13956.88 | 1983.71 | 0.889 | 14486.71 | 2658.80 | 13587.23 | 1230.90 | 0.522 |
| superiortemporal | 11073.67 | 1534.19 | 11072.33 | 1805.71 | 0.952 | 11047.06 | 1984.49 | 11059.95 | 1740.90 | 0.179 |
| supramarginal | 11162.16 | 1689.51 | 11252.45 | 2197.60 | 0.736 | 12062.35 | 2559.79 | 10629.00 | 1743.07 | 0.429 |
| frontalpole | 1411.56 | 201.69 | 1432.08 | 232.44 | 0.651 | 1469.41 | 220.29 | 1403.77 | 247.77 | 0.557 |
| temporalpole | 1913.79 | 451.74 | 1891.10 | 454.74 | 0.845 | 1905.41 | 525.17 | 1889.45 | 414.35 | 0.615 |
| transversetemporal | 1107.28 | 158.29 | 1166.98 | 215.95 | 0.140 | 1180.29 | 215.32 | 1166.50 | 221.18 | 0.945 |
| insula | 7317.12 | 1103.58 | 7249.63 | 1095.21 | 0.795 | 7514.71 | 1213.43 | 7059.68 | 1002.98 | 0.598 |

**Supplementary Table 1. Volume for each cortical parcellation.** Abbreviations: WM = white matter; lh = left hemisphere; rh = right hemisphere; VSS NM = visual snow syndrome patients without migraines; VSS M = visual snow syndrome patients with migraines.

| **T1 values (ms)** | **Controls** | **n = 43** | **VSS** | **n = 40** |  | **VSS NM** | **n = 17** | **VSS M** | **n = 22** |  |
| --- | --- | --- | --- | --- | --- | --- | --- | --- | --- | --- |
|  | Mean | SD | Mean | SD | *p*-values | Mean | SD | Mean | SD | *p*-values |
| **Left hemisphere** |  |  |  |  |  |  |  |  |  |  |
| bankssts | 1970.64 | 45.45 | 1940.69 | 50.35 | **0.006** | 1938.20 | 57.63 | 1939.42 | 43.99 | 0.941 |
| caudalanteriorcingulate | 1954.51 | 51.60 | 1922.62 | 43.58 | **0.003** | 1931.28 | 40.29 | 1915.07 | 46.40 | 0.260 |
| caudalmiddlefrontal | 1919.11 | 47.76 | 1882.84 | 55.39 | **0.002** | 1895.35 | 54.10 | 1872.22 | 56.59 | 0.205 |
| cuneus | 1822.89 | 41.15 | 1792.50 | 41.57 | **0.001** | 1794.01 | 37.00 | 1789.86 | 45.90 | 0.763 |
| entorhinal | 1923.43 | 84.89 | 1889.18 | 68.84 | 0.048 | 1892.11 | 71.89 | 1885.83 | 69.41 | 0.784 |
| fusiform | 1957.27 | 51.76 | 1910.79 | 62.04 | **<0.001** | 1916.43 | 61.10 | 1905.16 | 64.86 | 0.585 |
| inferiorparietal | 1921.12 | 42.72 | 1883.79 | 42.14 | **<0.001** | 1882.72 | 42.64 | 1883.60 | 43.45 | 0.950 |
| inferiortemporal | 1943.38 | 69.13 | 1912.66 | 72.47 | 0.051 | 1926.11 | 74.18 | 1900.75 | 72.20 | 0.289 |
| isthmuscingulate | 1788.04 | 42.56 | 1753.81 | 38.43 | **<0.001** | 1757.80 | 32.20 | 1748.55 | 42.43 | 0.460 |
| lateraloccipital | 1886.74 | 39.72 | 1848.81 | 40.60 | **<0.001** | 1849.23 | 44.08 | 1846.98 | 39.06 | 0.867 |
| lateralorbitofrontal | 1856.62 | 61.83 | 1830.32 | 48.77 | **0.035** | 1840.71 | 54.06 | 1820.11 | 43.40 | 0.195 |
| lingual | 1875.40 | 39.76 | 1828.85 | 40.96 | **<0.001** | 1829.10 | 41.59 | 1825.38 | 39.20 | 0.776 |
| medialorbitofrontal | 1911.23 | 65.34 | 1881.64 | 53.50 | **0.027** | 1884.42 | 56.92 | 1877.35 | 52.12 | 0.689 |
| middletemporal | 2009.60 | 58.01 | 1982.90 | 50.96 | **0.029** | 1989.24 | 62.33 | 1976.33 | 41.48 | 0.443 |
| parahippocampal | 1978.84 | 56.35 | 1929.83 | 54.05 | **<0.001** | 1934.24 | 51.91 | 1923.44 | 55.76 | 0.540 |
| paracentral | 1776.99 | 46.78 | 1745.79 | 49.17 | **0.004** | 1757.93 | 42.96 | 1735.79 | 53.40 | 0.171 |
| pars opercularis | 1963.23 | 55.29 | 1920.48 | 52.85 | **0.001** | 1929.88 | 58.19 | 1911.34 | 48.66 | 0.286 |
| pars orbitalis | 1943.99 | 55.15 | 1911.15 | 56.66 | **0.009** | 1916.05 | 62.77 | 1905.25 | 52.93 | 0.564 |
| pars triangularis | 1932.71 | 54.66 | 1895.04 | 50.25 | **0.002** | 1904.13 | 57.29 | 1886.72 | 44.85 | 0.294 |
| pericalcarine | 1802.47 | 34.89 | 1758.91 | 40.18 | **<0.001** | 1761.58 | 37.62 | 1754.70 | 42.32 | 0.601 |
| postcentral | 1846.69 | 44.60 | 1803.69 | 44.09 | **<0.001** | 1808.12 | 43.56 | 1799.16 | 45.81 | 0.540 |
| posteriorcingulate | 1885.54 | 47.16 | 1847.43 | 43.04 | **<0.001** | 1852.68 | 37.90 | 1842.87 | 47.92 | 0.493 |
| precentral | 1808.48 | 48.06 | 1788.04 | 60.06 | 0.090 | 1799.26 | 59.50 | 1779.10 | 61.76 | 0.311 |
| precuneus | 1885.50 | 43.01 | 1845.72 | 39.15 | **<0.001** | 1852.02 | 31.72 | 1839.46 | 44.30 | 0.330 |
| rostralanteriorcingulate | 1953.99 | 54.57 | 1926.88 | 46.60 | **0.018** | 1928.00 | 47.13 | 1925.20 | 48.19 | 0.857 |
| rostralmiddlefrontal | 1944.06 | 47.03 | 1910.59 | 46.58 | **0.002** | 1921.86 | 53.18 | 1900.74 | 40.50 | 0.167 |
| superiorfrontal | 1915.25 | 48.76 | 1881.45 | 46.88 | **0.002** | 1888.87 | 50.66 | 1874.81 | 44.91 | 0.365 |
| superiorparietal | 1851.78 | 44.40 | 1815.05 | 43.94 | **<0.001** | 1818.12 | 39.11 | 1810.91 | 48.19 | 0.619 |
| superiortemporal | 1980.96 | 46.30 | 1945.30 | 45.15 | **0.001** | 1951.45 | 46.72 | 1938.59 | 44.22 | 0.385 |
| supramarginal | 1945.93 | 44.33 | 1904.68 | 44.32 | **<0.001** | 1906.94 | 42.46 | 1901.38 | 46.93 | 0.704 |
| frontalpole | 1956.22 | 66.07 | 1944.58 | 54.03 | 0.384 | 1956.48 | 66.11 | 1934.66 | 43.14 | 0.221 |
| temporalpole | 1904.92 | 105.30 | 1852.74 | 108.88 | **0.029** | 1827.06 | 114.97 | 1870.88 | 104.88 | 0.223 |
| transversetemporal | 1805.44 | 49.77 | 1761.32 | 52.87 | **<0.001** | 1760.23 | 51.45 | 1761.06 | 56.08 | 0.962 |
| insula | 1880.32 | 45.69 | 1862.33 | 41.73 | 0.065 | 1869.64 | 46.00 | 1855.36 | 38.57 | 0.299 |
| **Right hemisphere** |  |  |  |  |  |  |  |  |  |  |
| bankssts | 1911.70 | 46.64 | 1890.25 | 41.93 | **0.031** | 1893.56 | 43.92 | 1886.20 | 41.44 | 0.595 |
| caudalanteriorcingulate | 1958.27 | 57.98 | 1931.94 | 41.00 | **0.020** | 1939.54 | 38.06 | 1925.73 | 43.92 | 0.310 |
| caudalmiddlefrontal | 1865.80 | 47.55 | 1833.50 | 47.34 | **0.003** | 1842.76 | 50.48 | 1825.50 | 45.46 | 0.270 |
| cuneus | 1797.67 | 40.72 | 1767.75 | 37.11 | **0.001** | 1762.65 | 32.42 | 1770.16 | 40.93 | 0.539 |
| entorhinal | 1854.44 | 82.68 | 1814.41 | 83.05 | **0.031** | 1810.83 | 98.71 | 1814.23 | 71.93 | 0.901 |
| fusiform | 1862.59 | 49.20 | 1831.95 | 44.79 | **0.004** | 1826.15 | 48.83 | 1833.74 | 41.38 | 0.602 |
| inferiorparietal | 1899.91 | 38.50 | 1874.57 | 38.35 | **0.004** | 1878.08 | 39.20 | 1870.86 | 38.91 | 0.570 |
| inferiortemporal | 1790.41 | 77.82 | 1752.92 | 74.81 | **0.028** | 1757.11 | 92.15 | 1749.89 | 62.44 | 0.772 |
| isthmuscingulate | 1738.16 | 47.34 | 1710.96 | 42.06 | **0.007** | 1724.11 | 35.41 | 1698.98 | 44.32 | 0.064 |
| lateraloccipital | 1854.97 | 34.95 | 1825.28 | 37.32 | **<0.001** | 1820.72 | 43.17 | 1827.13 | 32.91 | 0.601 |
| lateralorbitofrontal | 1945.90 | 56.88 | 1923.21 | 49.02 | 0.056 | 1926.41 | 49.05 | 1921.25 | 51.10 | 0.752 |
| lingual | 1818.18 | 38.97 | 1785.15 | 39.66 | **<0.001** | 1782.88 | 35.04 | 1783.93 | 42.14 | 0.934 |
| medialorbitofrontal | 1968.87 | 68.19 | 1948.88 | 52.83 | 0.141 | 1951.19 | 49.55 | 1946.37 | 57.36 | 0.784 |
| middletemporal | 1897.82 | 70.11 | 1868.15 | 46.11 | **0.027** | 1877.34 | 49.77 | 1861.54 | 44.05 | 0.300 |
| parahippocampal | 1910.21 | 54.96 | 1878.48 | 43.43 | **0.005** | 1884.57 | 43.13 | 1871.47 | 43.38 | 0.355 |
| paracentral | 1769.51 | 50.70 | 1738.70 | 49.56 | **0.006** | 1749.21 | 50.05 | 1729.69 | 49.60 | 0.233 |
| pars opercularis | 1915.78 | 44.93 | 1889.72 | 42.21 | **0.008** | 1891.63 | 39.73 | 1888.19 | 45.84 | 0.807 |
| pars orbitalis | 1966.90 | 59.73 | 1934.53 | 45.99 | **0.007** | 1938.52 | 52.73 | 1930.99 | 42.19 | 0.623 |
| pars triangularis | 1921.71 | 45.24 | 1897.54 | 40.45 | **0.012** | 1903.24 | 43.37 | 1891.99 | 38.95 | 0.400 |
| pericalcarine | 1761.83 | 38.82 | 1726.74 | 36.42 | **<0.001** | 1726.72 | 34.80 | 1724.14 | 37.09 | 0.826 |
| postcentral | 1790.10 | 40.77 | 1755.82 | 44.87 | **<0.001** | 1757.11 | 50.64 | 1754.21 | 42.11 | 0.847 |
| posteriorcingulate | 1865.01 | 47.72 | 1830.80 | 43.57 | **0.001** | 1839.61 | 37.67 | 1823.90 | 48.29 | 0.276 |
| precentral | 1760.52 | 46.44 | 1745.68 | 56.32 | 0.193 | 1755.14 | 55.22 | 1738.85 | 58.66 | 0.383 |
| precuneus | 1843.50 | 45.19 | 1808.07 | 37.10 | **<0.001** | 1814.86 | 31.51 | 1801.69 | 41.12 | 0.281 |
| rostralanteriorcingulate | 1969.71 | 67.82 | 1956.08 | 51.76 | 0.309 | 1965.90 | 33.03 | 1951.10 | 62.31 | 0.381 |
| rostralmiddlefrontal | 1937.25 | 49.41 | 1913.22 | 43.60 | **0.022** | 1917.68 | 47.40 | 1909.20 | 42.16 | 0.559 |
| superiorfrontal | 1919.69 | 47.03 | 1893.19 | 48.07 | **0.013** | 1901.90 | 50.17 | 1885.55 | 47.24 | 0.304 |
| superiorparietal | 1826.36 | 42.73 | 1794.28 | 39.36 | **0.001** | 1796.39 | 39.45 | 1792.64 | 41.05 | 0.776 |
| superiortemporal | 1895.14 | 44.83 | 1866.94 | 45.15 | **0.005** | 1863.66 | 42.80 | 1868.84 | 48.67 | 0.731 |
| supramarginal | 1895.43 | 40.76 | 1864.09 | 41.57 | **0.001** | 1866.89 | 49.05 | 1860.81 | 36.45 | 0.659 |
| frontalpole | 1935.88 | 67.23 | 1934.76 | 63.54 | 0.938 | 1941.65 | 73.64 | 1928.40 | 57.11 | 0.530 |
| temporalpole | 1829.43 | 142.81 | 1731.82 | 121.13 | **0.001** | 1713.32 | 114.43 | 1745.65 | 129.59 | 0.422 |
| transversetemporal | 1748.22 | 46.39 | 1711.84 | 46.06 | **0.001** | 1713.96 | 44.23 | 1709.16 | 49.12 | 0.754 |
| insula | 1867.75 | 39.12 | 1852.49 | 47.87 | 0.115 | 1849.78 | 54.91 | 1854.12 | 44.09 | 0.785 |

**Supplementary Table 2. T1 values for each cortical parcellation.** P-values that survived false discovery rate correction are marked bold. Abbreviations: lh = left hemisphere; rh = right hemisphere; VSS NM = visual snow syndrome patients without migraines; VSS M = visual snow syndrome patients with migraines.

| **Volumes (mm^3^)** | |  |  |  |  |  |  |  |  |  |
| --- | --- | --- | --- | --- | --- | --- | --- | --- | --- | --- |
|  | **Controls (n = 43)** | | **VSS (n = 40)** | |  | **VSS NM (n = 17)** | | **VSS M (n = 22)** | |  |
|  | Mean | SD | Mean | SD | *p*-values | Mean | SD | Mean | SD | *p*-values |
| AV | 120.30 | 17.39 | 121.36 | 19.81 | 0.720 | 122.69 | 17.77 | 120.89 | 21.90 | 0.213 |
| CeM | 59.90 | 7.24 | 60.92 | 8.25 | 0.504 | 60.94 | 7.84 | 60.84 | 8.92 | 0.348 |
| Cl | 28.75 | 4.69 | 30.01 | 6.97 | 0.290 | 29.61 | 5.58 | 30.26 | 8.13 | 0.277 |
| CM | 213.71 | 18.56 | 215.27 | 25.79 | 0.628 | 219.57 | 22.15 | 210.58 | 27.95 | 0.381 |
| LD | 20.97 | 4.66 | 25.02 | 7.55 | 0.003 | 23.50 | 6.45 | 26.14 | 8.42 | 0.116 |
| LGN | 254.45 | 28.16 | 257.56 | 28.05 | 0.560 | 266.37 | 25.53 | 249.82 | 28.61 | 0.324 |
| LP | 110.88 | 14.55 | 119.55 | 20.05 | 0.012 | 118.03 | 18.26 | 120.52 | 22.11 | 0.060 |
| L_Sg | 20.91 | 5.93 | 19.70 | 6.10 | 0.369 | 22.41 | 6.51 | 17.76 | 5.14 | 0.074 |
| MDI | 257.80 | 22.85 | 258.80 | 25.74 | 0.804 | 265.29 | 24.21 | 253.09 | 26.55 | 0.858 |
| MDm | 744.32 | 71.29 | 745.03 | 78.69 | 0.911 | 757.90 | 71.87 | 732.04 | 83.90 | 0.869 |
| MGN | 91.14 | 12.55 | 90.71 | 13.81 | 0.908 | 94.84 | 15.41 | 86.83 | 11.52 | 0.365 |
| MV_Re | 10.22 | 1.51 | 10.83 | 1.95 | 0.114 | 10.46 | 1.68 | 11.05 | 2.15 | 0.141 |
| Pf | 45.27 | 4.42 | 46.86 | 6.12 | 0.097 | 47.71 | 5.74 | 45.83 | 6.28 | 0.216 |
| PuA | 221.89 | 21.63 | 222.27 | 25.56 | 0.883 | 226.22 | 22.17 | 218.21 | 28.02 | 0.632 |
| Pull | 273.36 | 34.24 | 272.25 | 39.97 | 0.909 | 275.75 | 33.69 | 269.40 | 45.65 | 0.993 |
| PuL | 212.46 | 35.20 | 199.14 | 29.96 | 0.066 | 207.28 | 30.53 | 193.24 | 29.39 | 0.623 |
| PuM | 1171.15 | 105.50 | 1163.82 | 143.03 | 0.814 | 1180.84 | 110.01 | 1144.07 | 164.70 | 0.717 |
| VA | 387.64 | 44.91 | 389.63 | 54.59 | 0.784 | 404.85 | 49.89 | 378.49 | 57.54 | 0.793 |
| VAmc | 27.73 | 2.94 | 27.81 | 4.02 | 0.841 | 28.52 | 3.73 | 27.21 | 4.31 | 0.554 |
| VLa | 565.93 | 55.25 | 571.05 | 75.18 | 0.598 | 586.93 | 71.55 | 558.56 | 78.91 | 0.449 |
| VLp | 734.19 | 67.89 | 744.54 | 97.95 | 0.418 | 761.98 | 92.16 | 730.48 | 104.33 | 0.296 |
| VPL | 754.36 | 72.67 | 755.41 | 88.15 | 0.866 | 772.60 | 81.35 | 741.17 | 94.42 | 0.401 |

**Supplementary Table 3. Volumetrics thalamic nuclei.** Abbreviations**:** AV = anteroventral; CeM = central medial; Cl = central lateral; CM = centromedian; LD = laterodorsal; LGN = lateral geniculate; lh = left hemisphere; LP = lateral Posterior; L_Sg = suprageniculate; MDl = mediodorsal lateral parvocellular; MDm = mediodorsal medial magnocellular; MGN = medial geniculate; MV_Re = medial ventral reuniens; Pf = parafascicular; PuA = pulvinar anterior; PulI = pulvinar inferior; PuL = pulvinar lateral; PuM = pulvinar medial; rh = right hemisphere; VA = ventral anterior; VAmc = ventral anterior magnocellular; VLa = ventral lateral anterior; VLp = ventral lateral posterior, VPL = ventral posterolateral nucleus; VSS NM = visual snow syndrome patients without migraines; VSS M = visual snow syndrome patients with migraines.

| **T1 values (ms)** | |  |  |  |  |  |  |  |  |  |
| --- | --- | --- | --- | --- | --- | --- | --- | --- | --- | --- |
|  | **Controls (n = 43)** | | **VSS (n = 40)** | |  | **VSS NM (n = 17)** | | **VSS M (n = 22)** | |  |
|  | Mean | SD | Mean | SD | *p*-values | Mean | SD | Mean | SD | *p*-values |
| AV | 2914.24 | 145.99 | 2815.98 | 86.55 | **0.000** | 2836.41 | 89.04 | 2797.43 | 83.65 | 0.169 |
| CeM | 2809.80 | 110.57 | 2746.13 | 82.51 | **0.004** | 2763.51 | 98.33 | 2730.73 | 68.50 | 0.228 |
| Cl | 2933.47 | 370.01 | 2752.47 | 115.49 | **0.004** | 2749.50 | 83.70 | 2756.36 | 139.11 | 0.858 |
| CM | 2714.70 | 86.58 | 2658.59 | 69.15 | **0.002** | 2672.48 | 81.00 | 2645.33 | 58.25 | 0.231 |
| LD | 3055.01 | 392.89 | 2815.29 | 122.73 | **0.000** | 2858.69 | 140.02 | 2784.53 | 101.71 | 0.063 |
| LGN | 2742.64 | 116.89 | 2696.68 | 91.90 | 0.051 | 2691.97 | 56.39 | 2694.23 | 111.27 | 0.940 |
| LP | 2883.44 | 207.12 | 2740.51 | 92.47 | **0.000** | 2767.23 | 121.77 | 2721.12 | 59.79 | 0.129 |
| L_Sg | 2880.69 | 253.36 | 2835.02 | 227.64 | 0.392 | 2781.55 | 118.34 | 2858.89 | 275.04 | 0.286 |
| MDI | 2934.11 | 91.20 | 2881.47 | 78.88 | **0.006** | 2898.67 | 82.97 | 2865.88 | 75.47 | 0.205 |
| MDm | 3199.58 | 155.37 | 3103.43 | 110.69 | **0.002** | 3125.83 | 127.80 | 3084.65 | 97.39 | 0.260 |
| MGN | 2786.29 | 102.60 | 2728.17 | 78.62 | **0.005** | 2740.68 | 74.46 | 2713.45 | 79.45 | 0.283 |
| MV_Re | 2850.65 | 157.04 | 2753.91 | 127.05 | **0.003** | 2769.67 | 153.54 | 2737.90 | 105.86 | 0.449 |
| Pf | 2714.78 | 105.25 | 2658.25 | 97.12 | **0.013** | 2652.50 | 76.14 | 2658.86 | 112.85 | 0.843 |
| PuA | 2994.85 | 96.61 | 2929.89 | 80.29 | **0.001** | 2953.46 | 88.43 | 2910.46 | 71.65 | 0.102 |
| Pull | 2957.79 | 111.67 | 2904.63 | 96.49 | **0.023** | 2917.55 | 78.21 | 2889.67 | 108.11 | 0.376 |
| PuL | 2842.93 | 85.25 | 2769.64 | 78.03 | **0.000** | 2791.27 | 89.31 | 2753.10 | 67.56 | 0.137 |
| PuM | 3209.34 | 174.27 | 3091.85 | 97.75 | **0.000** | 3092.98 | 83.38 | 3089.26 | 111.23 | 0.909 |
| VA | 2656.78 | 93.59 | 2601.89 | 66.50 | **0.003** | 2619.09 | 73.23 | 2586.32 | 59.28 | 0.131 |
| VAmc | 2574.38 | 119.27 | 2523.45 | 105.14 | **0.043** | 2525.00 | 123.27 | 2523.69 | 94.36 | 0.970 |
| VLa | 2645.12 | 81.64 | 2597.03 | 68.31 | **0.005** | 2608.25 | 80.06 | 2586.19 | 58.73 | 0.327 |
| VLp | 2678.88 | 84.43 | 2625.23 | 69.55 | **0.002** | 2636.80 | 76.60 | 2614.30 | 64.72 | 0.327 |
| VPL | 2652.76 | 78.80 | 2600.95 | 65.62 | **0.002** | 2612.07 | 76.32 | 2590.04 | 56.67 | 0.307 |

**Supplementary Table 4. T1 values thalamic nuclei**. Abbreviations**:** AV = anteroventral; CeM = central medial; Cl = central lateral; CM = centromedian; LD = laterodorsal; LGN = lateral geniculate; lh = left hemisphere; LP = lateral Posterior; L_Sg = suprageniculate; MDl = mediodorsal lateral parvocellular; MDm = mediodorsal medial magnocellular; MGN = medial geniculate; MV_Re = medial ventral reuniens; Pf = parafascicular; PuA = pulvinar anterior; PulI = pulvinar inferior; PuL = pulvinar lateral; PuM = pulvinar medial; rh = right hemisphere; VA = ventral anterior; VAmc = ventral anterior magnocellular; VLa = ventral lateral anterior; VLp = ventral lateral posterior, VPL = ventral posterolateral nucleus; VSS NM = visual snow syndrome patients without migraines; VSS M = visual snow syndrome patients with migraines. P-values that survived FDR correction are marked bold.
